# A New First Break Picking for Three-Component VSP Data Using Gesture Sensor and Polarization Analysis

**DOI:** 10.3390/s17092150

**Published:** 2017-09-19

**Authors:** Huailiang Li, Xianguo Tuo, Tong Shen, Ruili Wang, Jérémie Courtois, Minhao Yan

**Affiliations:** 1 Fundamental Science on Nuclear Wastes and Environmental Safety Laboratory, Southwest University of Science and Technology, Mianyang 621010, China; shtlu06@126.com (T.S.); jeremiecourtois@swust.edu.cn (J.C.); yanminhao@swust.edu.cn (M.Y.); 2 Institute of Natural and Mathematical Sciences, Massey University, Auckland 0632, New Zealand; Ruili.wang@massey.ac.nz; 3 State Key Laboratory of Geohazard Prevention and Geoenvironment Protection, Chengdu University of Technology, Chengdu 610059, China

**Keywords:** first break picking, 3C VSP, gesture calibration, polarization analysis, AR-AIC

## Abstract

A new first break picking for three-component (3C) vertical seismic profiling (VSP) data is proposed to improve the estimation accuracy of first arrivals, which adopts gesture detection calibration and polarization analysis based on the eigenvalue of the covariance matrix. This study aims at addressing the problem that calibration is required for VSP data using the azimuth and dip angle of geophones, due to the direction of geophones being random when applied in a borehole, which will further lead to the first break picking possibly being unreliable. Initially, a gesture-measuring module is integrated in the seismometer to rapidly obtain high-precision gesture data (including azimuth and dip angle information). Using re-rotating and re-projecting using earlier gesture data, the seismic dataset of each component will be calibrated to the direction that is consistent with the vibrator shot orientation. It will promote the reliability of the original data when making each component waveform calibrated to the same virtual reference component, and the corresponding first break will also be properly adjusted. After achieving 3C data calibration, an automatic first break picking algorithm based on the autoregressive-Akaike information criterion (AR-AIC) is adopted to evaluate the first break. Furthermore, in order to enhance the accuracy of the first break picking, the polarization attributes of 3C VSP recordings is applied to constrain the scanning segment of AR-AIC picker, which uses the maximum eigenvalue calculation of the covariance matrix. The contrast results between pre-calibration and post-calibration using field data show that it can further improve the quality of the 3C VSP waveform, which is favorable to subsequent picking. Compared to the obtained short-term average to long-term average (STA/LTA) and the AR-AIC algorithm, the proposed method, combined with polarization analysis, can significantly reduce the picking error. Applications of actual field experiments have also confirmed that the proposed method may be more suitable for the first break picking of 3C VSP. Test using synthesized 3C seismic data with low SNR indicates that the first break is picked with an error between 0.75 ms and 1.5 ms. Accordingly, the proposed method can reduce the picking error for 3C VSP data.

## 1. Introduction

First break picking is a fundamental, but important step, in three-component (3C) vertical seismic profiling (VSP) data processing, such as velocity estimation, wave-field separation, and anisotropy estimates [1]. Any errors and/or misidentifications of these arrival times may have significant effects on the static corrections and velocity inversion [2], including the structural and lithological investigation employing passive seismic tomography methodologies [3]. During the last few decades, various techniques have been developed for determining first breaks automatically or semi-automatically [4], such as automatic methods available that are based on the correlation properties, on some statistical criteria, or on artificial neural networks for both individual and groups of traces [5]. These approaches have played a critical role in their different periods, respectively, especially the short-term average to long-term average (STA/LTA) and Akaike information criterion (AIC) algorithms are, nowadays, still the most widely used [6,7,8,9]. Additionally, the data quality also plays a significant role on the effectiveness of any picking algorithm [10]. However, they mainly focus on the condition that all geophones are in a regular state; in other words, their data can be directly used without rotation calibration processing, taking shallow seismic, microseismic, and three-dimension three-component (3D3C) seismic exploration, for instance. Consequently, it is probable that these approaches will not work properly when being directly applied to 3C VSP, due to the fact that the geophone’s layout is arbitrary when the 3C seismic explorations are applied in a borehole, which will cause the horizontal and vertical components of all geophones to change randomly [11,12]. Generally, a data calibration process is required to improve the quality of 3C data, which makes the 3C data uniform to a virtual reference direction using the azimuth and dip angle of geophones. Therefore, to accurately extract gesture information of geophones is crucial. Despite several sophisticated calibration approaches based on direct wave energy having been proposed to make this seismic data more regular, their calibration angles are obtained using the energy approximate evaluation algorithm with the original data, which is also not calibrated [13,14]. Hence, the authenticity and accuracy of data calibration is questionable. In this sense, it is not an easy task in many situations to identify the first arrival of 3C VSP, and a more suitable and accurate first break picking method needs to be developed for addressing this problem. 

In this paper, taking into account seismometer rotation randomness in the borehole, the gesture information of the geophone is obtained quickly, including the accurate dip and azimuth angle information, which is helpful in choosing a reasonable vibrator location and improving the seismic record’s signal-to-noise ratio (SNR). Depending on the hardware design, the gesture detection technology is integrated in the geophone, and the specific calibration procedure will be discussed in detail. After gaining a reasonable dataset, the improved autoregressive-Akaike information criterion (AR-AIC) (proposed by [15]) algorithm is chosen to identify these first arrivals combined with 3C VSP data characteristics. Moreover, the polarization analysis is adopted to constrain the scanning segment of AR-AIC, which will further enhance the picking accuracy of AR-AIC, and the constraint process is also introduced in detail. Finally, the contrast results between pre-calibration and post-calibration data are analyzed, which shows that the resolution of the calibrated waveform data improves significantly, the post-calibrated waveform is more regular, and the clear first break is convenient to pick. The improved picking algorithm using the maximum eigenvalue calculation of the covariance matrix is compared with STA/LTA and AR-AIC algorithms, and the validity of the proposed integrated picking method is also further verified by employing actual seismic data tests. Test results using synthesized 3C seismic datasets with low SNR indicate that the first break is picked with an error between 0.75 ms and 1.5 ms, actual 3C VSP data picking indicates that the proposed method can accurately estimate the first break, and the calculated demarcation layer thickness and velocity are more in accord with the actual geological layer. 

## 2. Rotation Calibration Algorithm Combined with Gesture Information 

### 2.1. Gesture Information Detection Method

The gesture information detection module provides an accurate dip angle and azimuth information measurement, which is widely used in quadrotor navigation. An inertial measurement unit (IMU) is arranged to achieve the gesture detection, which is an electronic device that measures and reports a craft’s velocity, orientation, and gravitational forces, using a combination of accelerometers and gyroscopes, and sometimes magnetometers, as well [16]. The sensor module used is a GY-85 (Sparkfun, Boulder, CO, USA) nine-axis degree of freedom (DOF) micro-electro-mechanical system (MEMS) IMU sensor, which is fixed at 10-bit resolution, with up to 13-bit resolution at ±16 g. Its supply voltage range is 2.0 V to 3.6 V, and has ultralow power consumption, as low as 23 μA in measurement mode. The size is only 2.2 cm × 1.7 cm, and values can be gathered using the I^2^C protocol. Its *X*-axis and *Y*-axis are horizontal and the *Z*-axis is vertical, which is consistent with the three components of the 3C VSP geophone. All these characteristics make it is suitable to detect the gesture information of the 3C VSP geophone. The accelerometer used on the GY-85 is the ADXL345 (Analog Devices, Norwood, MA, USA) from Analog Devices. It measures acceleration for all three axes (*x*, *y*, *z*) and has a resolution up to 13 bits (detecting changes less than 1.0°). The ADXL345 also has some nice extra features, like tap and double-tab detection, which can be used to trigger an interrupt on the Arduino. The GY-85 uses InvenSense’s ITG3200 (InvenSense, Sunnyvale, CA, USA) to measure orientation. It can sense motion on all three axes and the sensor values are digitized using a 16 bit ADC. Additionally, it also has an integrated temperature sensor. Honeywell’s HMC5883L (Honeywell, Morristown, NJ, USA) used in the GY-85 is a three-axis digital magnetometer. The chip is most commonly used as a digital compass to sense the angle from magnetic north (not true north) in degrees. The datasheet and code can be obtained in [17,18].

There are three attitude angles—vertical pitch, horizontal roll, and yaw—that can be obtained using the MEMS inertial sensor group. The pitch and roll angles can be measured by combining the data of the gyroscope and accelerometer, which correspond to the dip angle; the electronic compass can obtain the azimuth of the geophone by sensing the Earth’s magnetic field (yaw angle). Certainly, this module cannot be directly applied to 3C VSP geophones, due to the fact that geophones will be in a quiescent state under normal circumstances. The value of each axial component is distributed with the gravity acceleration ignoring external acceleration, which is different from quadrotor navigation which remains in a moving state. Additionally, the azimuth accuracy will be lowered when the electronic compass is either inclined or interfered with by the external magnetic field [19,20]. Consequently, taking the application occasion into account, the accelerometer can only be affected by seismic waves received from seismic sensors when a geophone is fixed, thus, the reliable output will be taken using gyroscope compensation [21,22]. Additionally, the appropriate tilt compensation to the electronic compass can be performed via the calculated inclination angle.

**Figure 1:**
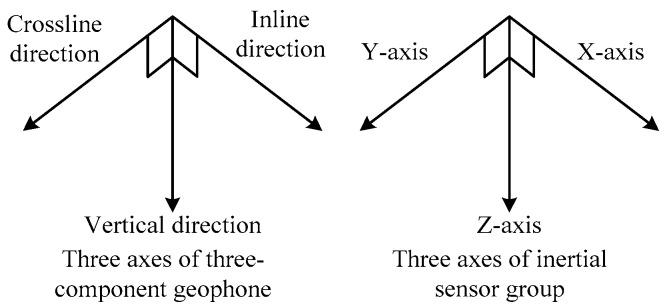
Combinations of 3C geophone and inertial sensors.

The gesture information detection module is used in connecting the integrated MEMS inertial sensor group coaxingly to the geophone’s three axial directions, as shown in Figure 1. Thus, the gesture information measurement can rapidly reflect the state of the 3C sensors, and this procedure will be achieved before seismic data acquisition. However, we do not consider the specific detection process in this paper, since many researchers have already done so [23,24].

### 2.2. Rotation Calibration Algorithm

**Figure 2:**
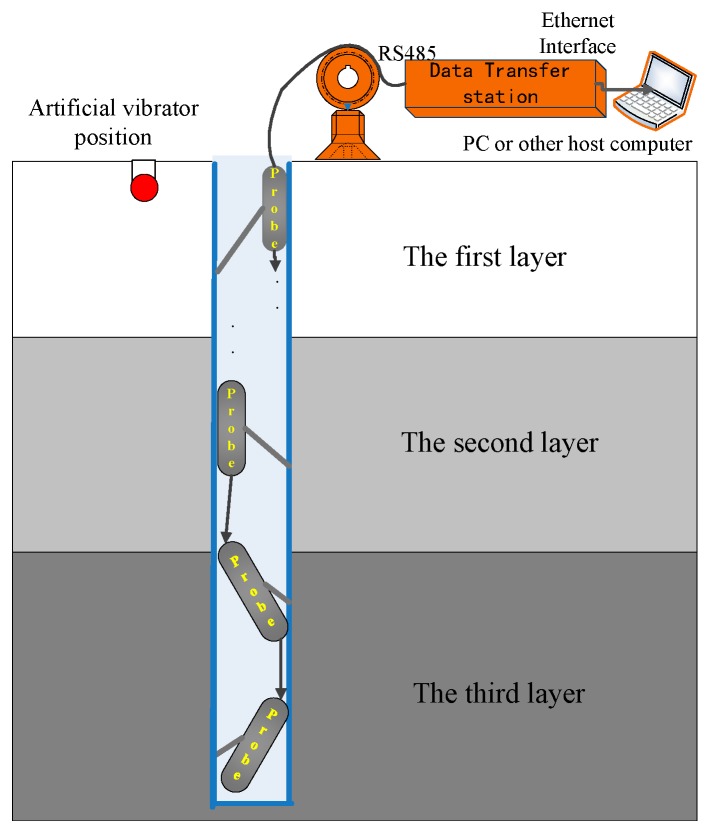
The 3C VSP scheme of the field construct.

**Figure 3:**
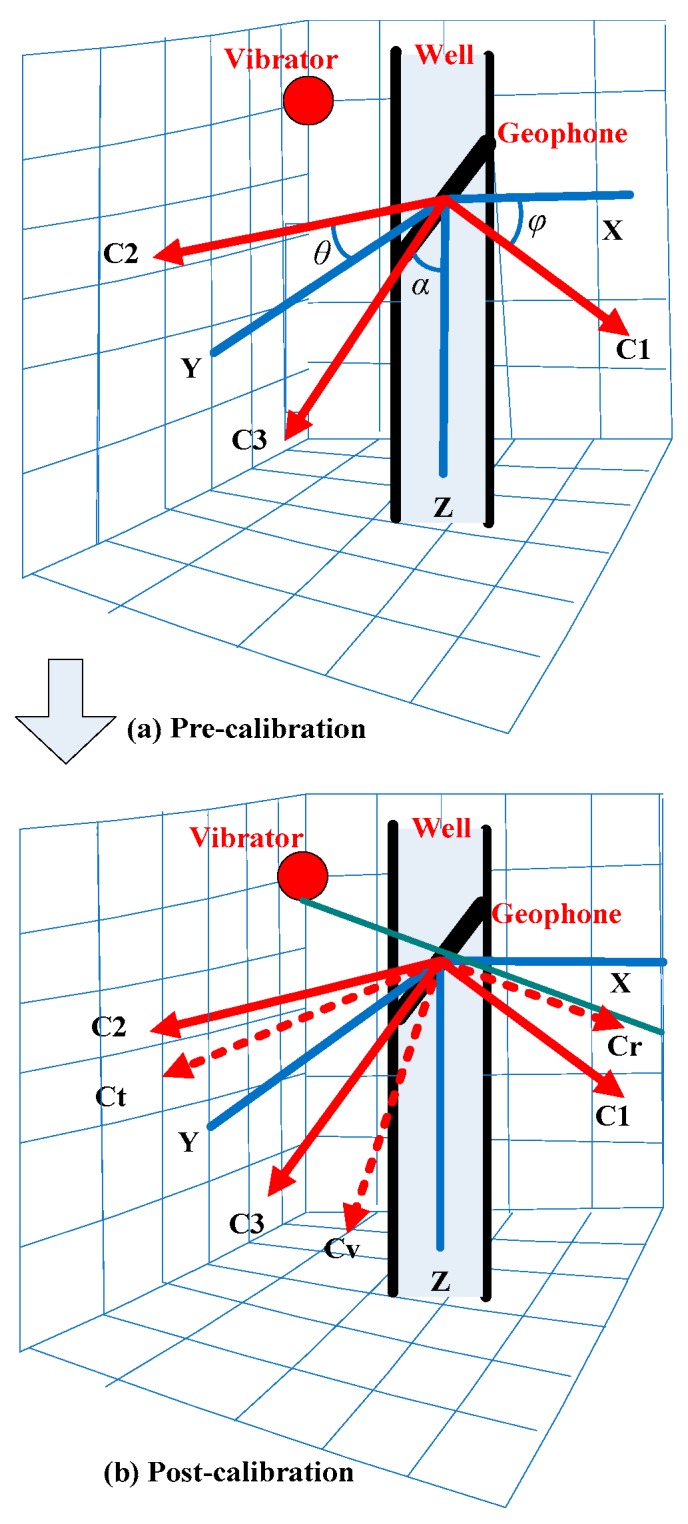
Diagram of the data calibration process.

The spatial position of the support arm for the borehole VSP probe is random after being opened when being placed in the downhole, as shown in Figure 2. Correspondingly, the spatial gesture of the geophone’s three components in the probe are also arbitrary, as shown in Figure 3a, *X*, *Y*, *Z* represent the geodetic coordinate system, and *c*1, *c*2, *c*3 refer to the spatial distribution of the downhole probe with actual measurements. Where *c*3 represents the vertical component, *c*1, *c*2 represent, respectively, the horizontal components; *c*1, *c*2 are perpendicular to each other, and they orient randomly between 0° and 360° in the inclined plane. Hence, it has a corresponding relationship between the geophone coordinate and the gesture detecting sensor, which means that the pitch angle *φ* represents the angle between the *X* axis to the *c*1 axis, the roll angle *θ* represents the angle between the *Y* axis to the *c*2 axis, and the tilt angle *α* represents the angle of *Z* axis to the *c*3 axis.

In the actual data calibration process, one of the horizontal components needs to be rotated in the direction that is uniform to the vibrator shot azimuth, as shown in Figure 3b. The rectangular coordinate system, after being rotated and oriented from three components of the geophone, are defined as *cr*, *ct*, *cv*, where the *cv* axis is plumb downward (vertical component), the *cr* axis is the horizontal azimuth which is consistent with the orientation of the vibrator position (radial component), and the *ct* axis is the remaining horizontal direction that is perpendicular to the *cr* axis (tangential component). In general, the 3C VSP dataset is mapped in the coordinate system of *c*1, *c*2, *c*3 after data acquisition; it can be said that it will rotate and project the VSP dataset from coordinate system *c*1, *c*2, *c*3 to *cr*, *ct*, *cv*. The specific calibration algorithm will be described below.

*c*1, *c*2, *c*3 are set as data records for the 3C VSP geophone, and when rotating the three components to the *X*, *Y*, *Z* coordinate system, the result is as follows:

(1) $$\left[\begin{array}{c} x\\ y\\ z \end{array}\right]=\left[\begin{array}{ccc} \tan\varphi & 0 & 0\\ 0 & \tan\theta & 0\\ 0 & 0 & \cos\alpha \end{array}\right]\left[\begin{array}{c} c1\\ c2\\ c3 \end{array}\right]$$

In the *X*, *Y*, *Z* coordinate system, (*xS*, *yS*, *zS*) is set as the coordinate position of the vibrator, which can be directly measured and recorded using a geological compass before constructing, assuring the angle is relative to the geodetic coordinate system, and (*xR*, *yR*, *zR*) is set as the coordinate position of the geophone. To facilitate the mathematical analysis, the *cr*, *ct*, *cv* coordinate system that has been calibrated from the 3C geophone is considered as the result of the *X*, *Y*, *Z* coordinate system rotation. That is, if keeping the *X* axis stationary first, then the *Y* axis and *Z* axis are rotated with the angle A around the *X* axis in a plane that is perpendicular to the *X* axis. Thus, an intermediate transitional coordinate system *x*, *y*′, *z*′ can be obtained, and the mathematical relationship between it and *X*, *Y*, *Z* coordinate system is:

(2) $$\left[\begin{array}{c} x\\ y\prime \\ z\prime \end{array}\right]=\left[\begin{array}{ccc} 1 & 0 & 0\\ 0 & \cos A & \sin A\\ 0 & -\sin A & \cos A \end{array}\right]\left[\begin{array}{c} x\\ y\\ z \end{array}\right],$$

where cos *A* and sin *A* can be calculated as:

(3) $$\cos A=\frac{yR-yS}{\sqrt{{\left(yR-yS\right)}^{2}+{\left(zR-zS\right)}^{2}}},$$

(4) $$\sin A=\frac{zR-zS}{\sqrt{{\left(yR-yS\right)}^{2}+{\left(zR-zS\right)}^{2}}},$$

Similarly, keeping the *y*′ axis stationary, *x*, *z*′ are rotated with angle B around the *y*′ axis on a plane perpendicular to the *y*′ axis; thus, an intermediate transitional coordinate system *x*′, *y*′, *cv* can be obtained, and the following mathematical relationship between it and the *x*, *y*′, *z*′ coordinate system occurs:

(5) $$\left[\begin{array}{c} x\prime \\ y\prime \\ \mathrm{cv} \end{array}\right]=\left[\begin{array}{ccc} \cos B & 0 & -\sin B\\ 0 & 1 & 0\\ \sin B & 0 & \cos B \end{array}\right]\left[\begin{array}{c} x\\ y\prime \\ z\prime \end{array}\right],$$

where cos *B* and sin *B* can be calculated as:

(6) $$\cos B=\frac{xR-xS}{\sqrt{{\left(xR-xS\right)}^{2}+{\left(zR-zS\right)}^{2}}},$$

(7) $$\sin B=\frac{zR-zS}{\sqrt{{\left(xR-xS\right)}^{2}+{\left(zR-zS\right)}^{2}}},$$

Finally, keeping the *cv* axis stationary, and *x*′, *y*′ rotated with angle C around the *cv* axis on a plane perpendicular to the *cv* axis; thus, the final calibration coordinate system *cr*, *ct*, *cv* can be obtained, and the mathematical relationship between it and *x*′, *y*′, *cv* coordinate system is:

(8) $$\left[\begin{array}{c} cr\\ ct\\ cv \end{array}\right]=\left[\begin{array}{ccc} \cos C & \sin C & 0\\ -\sin C & \cos C & 0\\ 0 & 0 & 1 \end{array}\right]\left[\begin{array}{c} x\prime \\ y\prime \\ cv \end{array}\right],$$

where cos *C* and sin *C* can be calculated as:

(9) $$\cos C=\frac{xR-xS}{\sqrt{{\left(xR-xS\right)}^{2}+{\left(yR-yS\right)}^{2}}},$$

(10) $$\sin C=\frac{yR-yS}{\sqrt{{\left(xR-xS\right)}^{2}+{\left(yR-yS\right)}^{2}}},$$

In all, the function relation between the coordinate system *cr*, *ct*, *cv* and the *X*, *Y*, *Z* coordinate system can be obtained combing with Equations (2), (5) and (8):

(11) $$\begin{array}{l} \left[\begin{array}{c} cr\\ ct\\ cv \end{array}\right]=\left[\begin{array}{ccc} \cos C & \sin C & 0\\ -\sin C & \cos C & 0\\ 0 & 0 & 1 \end{array}\right]\cdot \\ \left[\begin{array}{ccc} \cos B & 0 & -\sin B\\ 0 & 1 & 0\\ \sin B & 0 & \cos B \end{array}\right]\left[\begin{array}{ccc} 1 & 0 & 0\\ 0 & \cos A & \sin A\\ 0 & -\sin A & \cos A \end{array}\right]\left[\begin{array}{c} x\\ y\\ z \end{array}\right] \end{array},$$

Putting Equation (11) into Equation (1), the result will be:

(12) $$\begin{array}{l} \left[\begin{array}{c} cr\\ ct\\ cv \end{array}\right]=\left[\begin{array}{ccc} \cos C & \sin C & 0\\ -\sin C & \cos C & 0\\ 0 & 0 & 1 \end{array}\right]\left[\begin{array}{ccc} \cos B & 0 & -\sin B\\ 0 & 1 & 0\\ \sin B & 0 & \cos B \end{array}\right]\cdot \\ \left[\begin{array}{ccc} 1 & 0 & 0\\ 0 & \cos A & \sin A\\ 0 & -\sin A & \cos A \end{array}\right]\left[\begin{array}{ccc} \tan\varphi & 0 & 0\\ 0 & \tan\theta & 0\\ 0 & 0 & \cos\alpha \end{array}\right]\left[\begin{array}{c} c1\\ c2\\ c3 \end{array}\right] \end{array},$$

Equation (12) is the final calibration formula of the 3C VSP dataset. The pitch angle *φ*, roll angle *θ,* and tilt angle *α* of the 3C geophone in Equation (12) can be obtained using the gesture detection module.

## 3. Improved AR-AIC Algorithm Using Polarization

### 3.1. AR-AIC Algorithm

It is well known that the most commonly developed algorithm is the autoregressive (AR) model using the AIC as a statistical method. Approaches using the AR model are based on the assumption that seismic datasets can be approximately divided into two locally-stationary sequences (normally, noise and signal) determined according to the AR process, and the point for the minimum value of AIC is defined as the first break [10,25,26,27]. For the sample point k of a seismic dataset of length *N*, the AIC value can be calculated as Equation (13):

(13) $$\mathrm{AIC}(k)=(k-M)\log(\sigma_{1,\max}^{2})+(N-M-k)\log(\sigma_{2,\max}^{2})+C,$$

where *M* is the order of an AR model fitting the datasets, $\sigma_{1,\max}^{2}$ and $\sigma_{2,\max}^{2}$ are the variances of two 3C VSP data segments (from *M* + 1 to *k* and form *k* + 1 to *N* − *M*), and *C* is a constant [28]. In order to obtain the AIC values, the AR order needs to be estimated via repeated experiments and corrections, and the AR coefficients can be calculated by adopting the Yule-Walker function. It needs a larger amount of computation as a result of the matrix inversion operation when a high-order AR exists [2,9]. Consequently, [15] proposed a simplified form of the AIC function directly from the seismic datasets ignoring the AR coefficients, and the improved AIC (called M-AIC) function is defined as:

(14) AIC(k)=k×lg(var(x[1,k]))+(N−k−1)×lg(var(x[(k+1),N]),

where *k* corresponds to all samples in the *x* time sequences, var(x[1,k]) and var(x[(k+1),N] represent, respectively, the variance of two locally-stationary time series. Considering the efficiency and complexity of the calculation, the M-AIC will be selected as the picking algorithm in this paper.

Certainly, the M-AIC adopts variant-window lengths to scan the whole range of a given time window (TW) of seismic sequences, which makes the global minimum point the first break. Given a seismic dataset including a clear first break, the M-AIC algorithm can perform an accurate picking, however, it is worth mentioning that several local minimum values will appear when being applied to seismic datasets with low signal to noise ratios (SNR), and there is always a global minimum point in a time window, which will be taken as the first break without checking for the existence of the valid seismic signals in the datasets, whereas the M-AIC algorithm should be improved to adapt 3C VSP datasets with low SNR and optimize the picking accuracy.

### 3.2. Polarization Analysis

According to the feature of the M-AIC picker, its picking accuracy will be affected when noise signals exist in seismic datasets. Therefore, a method which focuses on reducing the influence of the noise signal will be developed without using the conventional digital filter, since the original signal will be more or less affected by any digital filters. Here we will use the covariance matrix to constrain the scanning segment of the M-AIC picker, which combines with the polarization attributes of the 3C seismic data. They have different polarization characteristics to valid seismic datasets and noise signals, such as the common seismic recordings having an ellipsoidal polarization trajectory. However, in the noisy datasets there exist irregular polarization trajectories, sometimes even having no polarization. 

A covariance matrix for the polarization analysis assume that the data are composed of three orthogonal seismic recordings corresponding to the east, north, and vertical components (corresponding respectively to *X*, *Y* and *Z*) [29,30]. The covariance matrix can be computed as Equation (15) using a T-samples sliding time window (the choice of time window length was suggested by [2], including subsequent calculations).

(15) $$M_{i}=\left[\begin{array}{ccc} \mathrm{var}(X) & \mathrm{cov}(X,Y) & \mathrm{cov}(X,Z)\\ \mathrm{cov}(X,Y) & \mathrm{var}(Y) & \mathrm{cov}(Y,Z)\\ \mathrm{cov}(X,Z) & \mathrm{cov}(Y,Z) & \mathrm{var}(Z) \end{array}\right],$$

in which *M_i_* is the total energy in the selected time window, cov(X,Y) and var(X) represent, respectively, the covariance between any two components *X* and *Y* or *X* and *X*, the same as the others, which are calculated as:

(16) $$\begin{array}{l} \mathrm{cov}(X,Y)=\frac{1}{T}\overset{T_{2}}{\underset{i=T_{1}}{\sum }}(x_{i}-\bar{x})(y_{i}-\bar{y})\\ \mathrm{var}(X)=\frac{1}{T}\overset{T_{2}}{\underset{i=T_{1}}{\sum }}{\left(x_{i}-\bar{x}\right)}^{2} \end{array},$$

where $\bar{x}$, $\bar{y}$, and $\bar{z}$ are defined as:

(17) $$\left\{ \begin{array}{l} \bar{x}=\frac{1}{T}\overset{N_{2}}{\underset{i=N_{1}}{\sum }}x_{i}\\ \bar{y}=\frac{1}{T}\overset{N_{2}}{\underset{i=N_{1}}{\sum }}y_{i}\\ \bar{z}=\frac{1}{T}\overset{N_{2}}{\underset{i=N_{1}}{\sum }}z_{i} \end{array}\right.,$$

To achieve the relevant calculation for *M_i_*, the corresponding eigenvalues (*λ*_1_ > *λ*_2_ > *λ*_3_) and eigenvector matrix *u* = (*u*1, *u*2, *u*3) of *M_i_* can be obtained, which can be used to demonstrate the energy and direction of particle motion [29,31]. This means that the three eigenvalues are usually nonzero, and the energy of the seismic signal is mainly focused on the maximum eigenvalue *λ*_1_ [31], which will be adopted to the improved algorithm. Using the eigenvalues (*λ*_1_, *λ*_2_, and *λ*_3_, respectively), two characteristic functions (*CF*) can be estimated as:

(18) $$CF_{1}(i)=1-\frac{\lambda_{2}(i)}{\lambda_{1}(i)},$$

(19) $$CF_{2}(i)=1-\frac{2\times \lambda_{3}(i)}{\lambda_{1}(i)+\lambda_{2}(i)},$$

**Figure 4:**
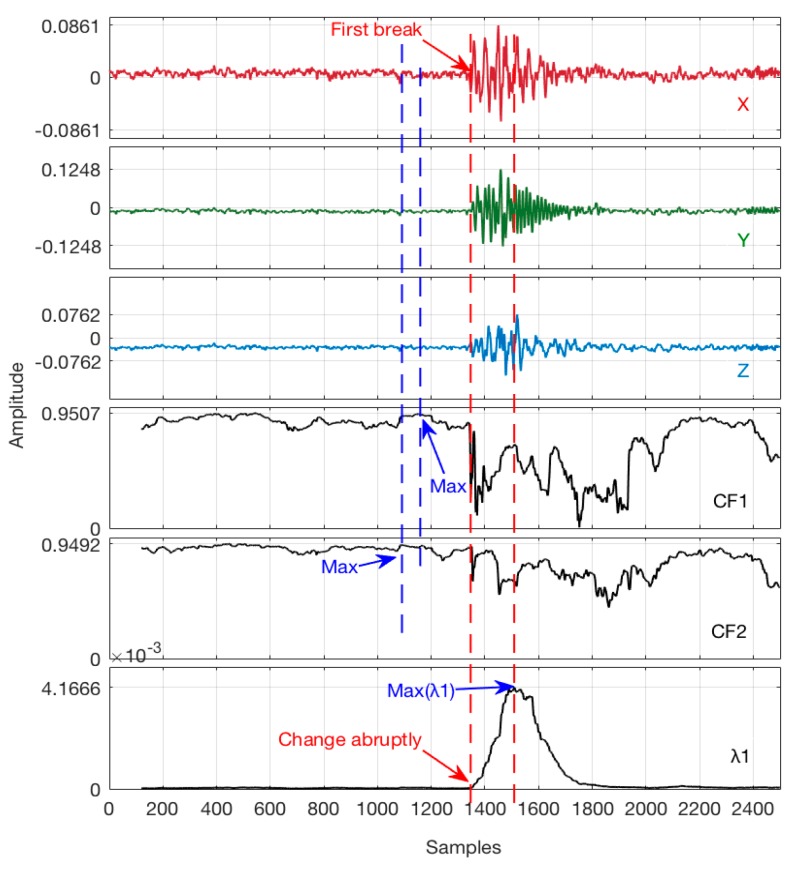
Picking comparison of *λ*_1_, *CF*_1_, and *CF*_2_ using actual data (TW = 120 samples for *λ*).

It is generally considered that the maximum value point of *CF*1 is the first arrival of the P-wave due to its linear polarization characteristic, and the maximum value point of *CF*2 is the first arrival of the S-wave [31,32,33]. However, this theory may be questionable due to the fact that the eigenvalues of the covariance matrix mainly reflects the energy within the time window, which changes along with the amplitude of the seismic waveform particle motion. As a result, the maximum value point of *CF*1 should not be taken as the first arrival, but these approaches have provided sufficient support to use the eigenvalue of the covariance matrices. It may be more reasonable that the first break is picked in the point while the eigenvalue *λ*_1_ changes abruptly [34]. Given an actual 3C seismic record, a basic comparison test was performed to demonstrate the difference of *λ*_1_, *CF*_1_ and *CF*_2_, as shown in Figure 4, and it is apparent that the eigenvalue *λ*_1_ can be used as a constrained criterion to the M-AIC picking algorithm.

It is further confirmed that the above picking criteria may be improper in some circumstances in Figure 4. However, it shows a good principle to set a constrained scanning segment utilizing the change trajectory of eigenvalue *λ*_1_, which can respond reasonably to valid seismic records.

### 3.3. Improved Algorithm

Since M-AIC can be regarded as an effective picker with high data quality, consequently, careful selection of scanning segment is extremely important to achieve better results adopting the M-AIC algorithm. Depending upon the characteristic of the M-AIC, the maximum eigenvalue *λ*_1_ is recommended to determine the scanning segment of the M-AIC which can improve its suitability with low SNR signals. This workflow can be summarized as follows:

(1) Selecting the scanning segment. Choosing the point which is two time windows length earlier than the location of the largest eigenvalue *λ*_1_ as the starting point by means of polarization analysis based on covariance matrix calculation, the eigenvalue *λ*_1_ can be determined based on the slope. Correspondingly, it will be seen as the end point when *λ*_1_ reaches its maximum.
(2) First break picking. Directly applying the M-AIC algorithm to the established seismic data segment via step (1), then choosing the global minimum as the first break.

**Figure 5:**
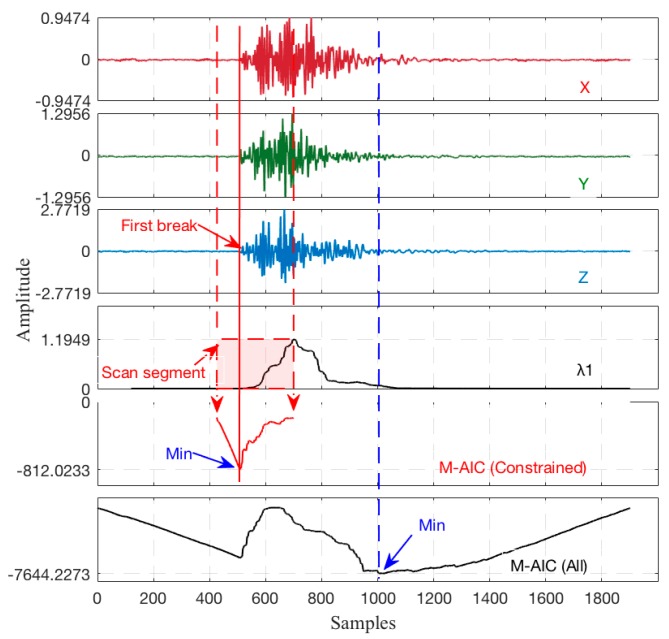
Picking comparison of all M-AIC and M-AIC with a constrained scanning segment using actual data (TW = 120 samples for *λ*_1_).

To demonstrate the performance and to check the efficiency of the improved method, a typical experiment was carried out using actual field datasets (considering the recommended time window length). As we can observe from the Figure 5, the minimum value of M-AIC, which calculates the selected data segment (red region) using eigenvalue *λ*_1_, can indicate a distinct first break, however, the minimum value of all M-AIC misses accurately exhibiting the presence of a first arrival. 

## 4. Tests and Discussion

### 4.1. Rotation Calibration Comparison Test

**Figure 6:**
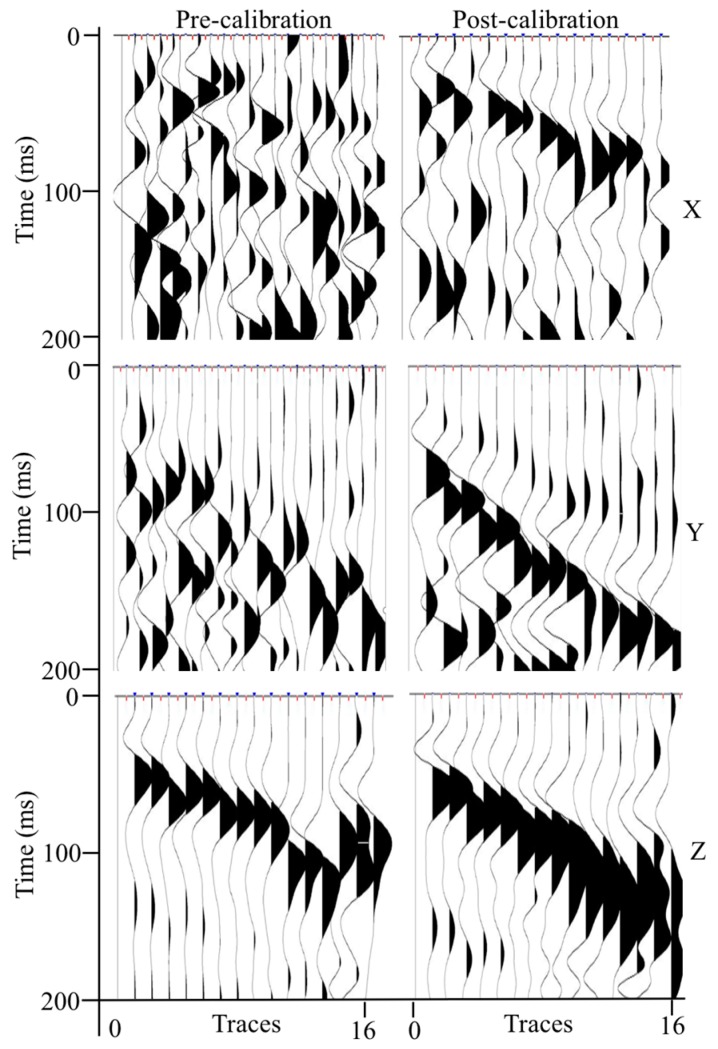
Contrast waveform of trace gathering (*X* is the radial component, *Y* is the tangential component, and *Z* is the vertical component).

Given the field 3C VSP data, a comparison test between pre-calibration and post-calibration process was performed to verify the application effects of rotation calibration using gesture detection technology. For illustrative purposes, the trace gathering procedure was implemented to the 3C VSP data, which means each component was shown separately. Considering space limitations, only the gathered results of the vibrator located 1 m to the left are given in Figure 6. 

As shown in Figure 6, the pre-calibration waveform of *X* and *Y* components are irregular, and cannot reflect a clear VSP record; in contrast, the post-calibration waveform of *X* and *Y* components can demonstrate a more regular seismic event. Certainly, they have a slight difference between the pre-calibration and post-calibration waveform of the *Z* components, this may be due to the fact that the downhole probes are close to vertical, but the post-calibration waveform should be more reliable from the result. Practically, it can significantly achieve a better seismic record adopting rotation calibration, which can help to precisely perform calculation the eigenvalues of covariance matrices when adopting 3C seismic data, moreover, and the first breaks of post-calibration waveforms are also convenient to pick manually.

### 4.2. Picking Algorithm Test

**Figure 7:**
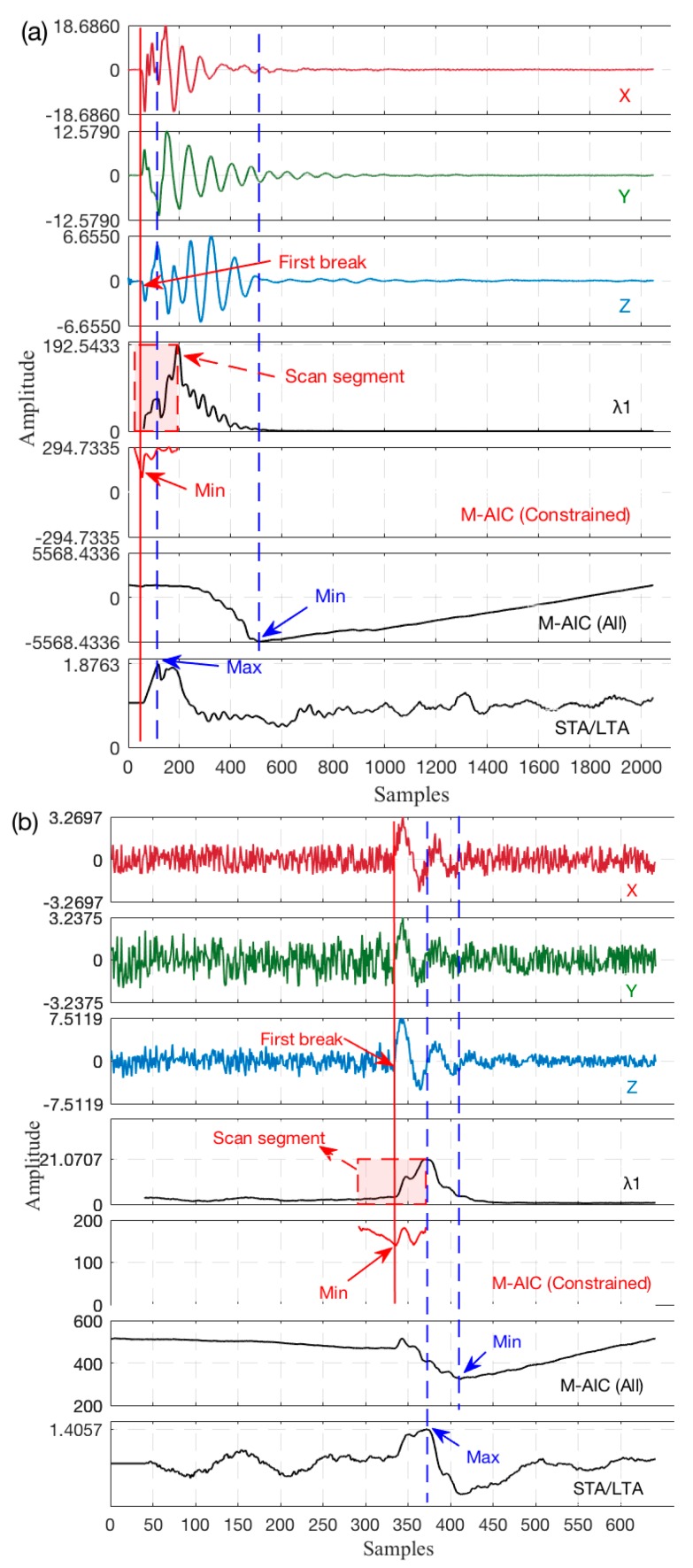
Picking comparison among the improved M-AIC, M-AIC, and STA/LTA algorithms. (**a**) Actual 3C VSP data (TW = 30 samples for λ, STA TW = 30 samples, LTA TW = 120 samples); and (**b**) synthesized 3C seismic data (TW = 120 samples for λ, STA TW = 60 samples, LTA TW = 180 samples).

To further demonstrate the performance of the improved first break picking algorithm, we use actual 3C VSP data sets and synthesized 3C seismic datasets with low SNR (dominant frequency of P-wave is 100 Hz, 0.25 ms sample interval and synthetic noise account for 30%) to carry out a *Z*-component (it should be mainly composed of P-wave energy) picking comparison test among the proposed method, M-AIC, and the STA/LTA picker (considering the recommended time window length). The results of this process for the three catalogs are shown in Figure 7.

Analyses of the results in Figure 7 indicate that the proposed method can accurately estimate the first break when being applied to actual datasets. However, all of them obtain an improper picking result for traditional M-AIC and STA/LTA algorithms, and there are several factors that could account for the missing *Z*-component arrivals, such as the M-AIC picker defining its global minimum point as the onset point ignoring the multiple arrivals and noise signals in a long time sequence, and it is necessary to provide a constrained estimate of scanning space in the case of better accuracy [28]. As for the STA/LTA picker, it intensively depends on the selection of long and short time windows, but the STA and LTA window sizes depend on the frequency characteristics of the seismic waveform; consequently, the window sizes of the STA/STA must change dynamically along with various seismic waveform characteristics [35], which may be not be suitable to achieve a precise picking in the conventional condition.

### 4.3. Field Application Test

**Figure 8:**
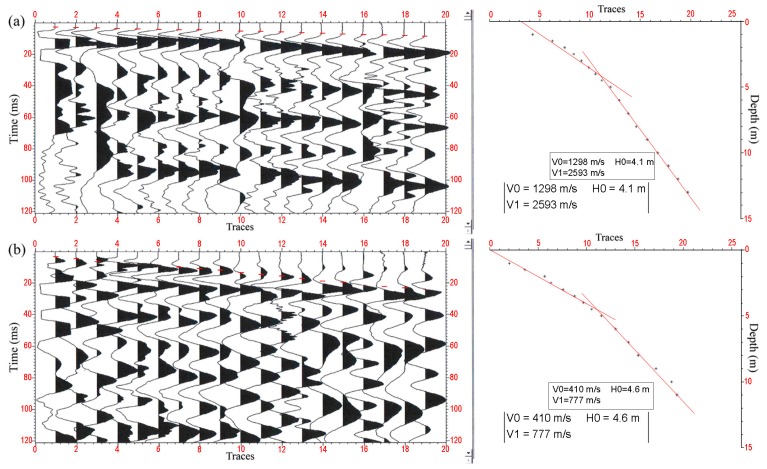
The calibration processing comparison using *Z*-component. (**a**) shows the post-calibration; (**b**) shows the pre-calibration, and the red lines were obtained from the interconnection with the first break picking point of the 3C VSP waveform, which can reflect the different layer velocity.

In order to perform stronger tests on the reliability of the improved algorithm we apply it to field 3C VSP exploration. As indicated by Figure 8, it can be seen from the picking results of the two trace gathers that the first arrivals without calibration are irregular in Figure 8b, and the geological layering calculation result using picked breaks is 4.6 m, which tends to be somewhat greater compared to the calibrated calculation result, but its calculated velocity cannot be determined, and the indicated result in Figure 8b is not distinctly consistent to field circumstances. However, as shown in Figure 8a, the trend of the first break with post-calibration is normal, and the calculated thickness of the fitting demarcation layer is 4.1 m, which is more in accord with the actual geological layer, and the calculated velocity is also more reasonable.

## 5. Conclusions

Considering the unreliability of the first break picking when 3C VSP is applied in a borehole, due to the randomness of the 3C VSP geophone state and the complexity of the construction environment when being applied, we have proposed a new approach to process 3C VSP data aimed to automatically identify and pick the first break in this paper. The processing is carried out in three steps. First, a gesture detection technique borrowed from quadrotor navigation is integrated to rapidly measure the azimuth and dip angle of the downhole geophone, which is also helpful to choose the vibrator position. Subsequently, the rotation calibration method is performed to make the 3C data be re-projected with respect to the vibrator shot orientation, which can enhance the quality and reliability of the original 3C VSP data. The second stage addresses a thorny problem of the scanning space restriction for the M-AIC picker, and polarization analysis based on calculation eigenvalues of covariance matrices for 3C seismic data is employed to constrain the scanning segment of seismic time sequences. Using the eigenvalues series, the point when eigenvalue changing abruptly and maximum eigenvalue are adopted to determine the start and end point of scanning segment, which can ensure a proper calculation space to M-AIC picker. Finally, the M-AIC algorithm is used to estimate the first break using its global minimum point under the limitative space, thus leading to quality enhancement of the picked arrivals. 

Testing the rotation calibration method on field 3C VSP datasets indicates that it can reliably enhance the quality of original seismic recordings. In other words, it can achieve a more regular seismic record, which can help to precisely perform first break picking automatically and manually. In contrast to traditional M-AIC and STA/LTA approaches, results after applying the improved picking algorithm to poor-quality field 3C data examples demonstrate that the improved M-AIC picker can achieve a robust and accurate identification Moreover, field 3C VSP exploration was carried to further confirm the effectiveness and reliability of the proposed combined method, and we believe that the integrated method can be utilized with a relatively high confidence to process 3C VSP data. Experiments using synthesized 3C seismic data with low SNR illustrate that it can achieve an error between 0.75 ms and 1.5 ms, and it also further indicates that the proposed method can deduce the picking error for 3C VSP data.
